# Flexibility on a Firm Foundation—The Central Electricity Generating Board’s Promotion of Nuclear Power in Oldbury-on-Severn

**DOI:** 10.1007/s00048-026-00445-9

**Published:** 2026-03-03

**Authors:** Christian Götter

**Affiliations:** TU Braunschweig, Braunschweig, Germany

**Keywords:** Nuclear Power, Atomic Energy, Acceptance, Technology, Britain, Industry Communication, Kernkraft, Atomenergie, Akzeptanz, Technologie, Großbritannien, Industriekommunikation

## Abstract

In March 1959, the small parish of Oldbury-on-Severn in Gloucestershire was singled out to become the site of one of the last reactors in the United Kingdom’s first “generation” of atomic power plants. The initial reception was ambiguous. Local people feared the loss of land, of traditional fishing rights in the nearby River Severn, and radioactive fallout. In unison, the parish council rejected the project, but accepted that the majority of Thornbury Rural District, to which it belonged, welcomed it as something that was in the national interest, was deemed safe by experts, and was a source of pride. In May, an exhibition on nuclear energy was opened in Thornbury to convince local people of the technology’s benefits. It was a first step in a long row of measures taken to argue for the power station and its positive contribution to the area. This paper demonstrates that the Central Electricity Generating Board (CEGB) and with it, the British state, as an early adopter of nuclear power, never ceased to fight for the technology’s acceptance, but faced challenges ranging from initial local skepticism to transnational catastrophes such as Chernobyl. In doing so, the CEGB resorted to a variety of measures, from the initial exhibition to large open days, and profited from institutionalized ones, namely public inquiries and a “Local Liaison Committee” functioning as a hinge between the power plant and its neighborhood. The paper thereby uncovers the often mundane practices by which technology acceptance was produced and maintained on the ground.

## Abstract (de)

Im März 1959 wurde die kleine Gemeinde Oldbury-on-Severn in Gloucestershire als Standort für einen der letzten Reaktoren der ersten ‚Generation‘ von Atomkraftwerken im Vereinigten Königreich ausgewählt. Die ersten Reaktionen waren zwiegespalten. Die lokale Bevölkerung befürchtete den Verlust von Land, von traditionellen Fischereirechten im nahe gelegenen Severn und radioaktiven Fallout. Der Gemeinderat lehnte das Projekt ab, akzeptierte aber, dass die Mehrheit des Thornbury Rural District, zu dem sie gehörte, es als etwas begrüßte, das von nationalem Interesse war, von Experten als sicher eingestuft wurde und etwas war, auf das man stolz sein konnte. Im Mai wurde in Thornbury eine Ausstellung über Kernenergie eröffnet, um die Menschen vor Ort von den Vorteilen der Technologie zu überzeugen. Sie war ein erster Schritt in einer langen Reihe von Maßnahmen, die für das Kraftwerk werben und es als positiv für die Region darstellen sollten. Dieser Aufsatz zeigt, dass das Central Electricity Generating Board, mithin der britische Staat als Vorreiter in der kommerziellen Nutzung der Kernenergie, immer wieder um die Akzeptanz der Technologie kämpfte. Dabei sah es sich mit Herausforderungen konfrontiert, die von anfänglicher, lokaler Skepsis bis hin zu transnationalen Katastrophen wie Tschernobyl reichten. Deshalb griff es auf eine Vielzahl von Maßnahmen zurück, von der genannten Ausstellung bis hin zu volksfestartigen Tagen der offenen Tür. Auch profitierte es von institutionalisierten Maßnahmen, namentlich der Tradition der „Public Inquiries“ und einem „Local Liaison Committee“, das als Scharnier zwischen dem Kraftwerk und seiner Umgebung fungierte. Der Aufsatz demonstriert dabei die oft profanen Praktiken, mit denen die Akzeptanz der Technologie vor Ort hergestellt und aufrechterhalten wurde.

## Introduction: Accountable, Transparent, Prestigious, and Familiar. The Central Electricity Generating Board’s Practice of Making a Nuclear Power Plant Acceptable

In Britain, so the usual story goes, nuclear energy was hardly controversial, especially if one compares the technology’s level of acceptance there with that in Germany (Oberloskamp 2022: 418–19). This impression, however, not only ignores oftentimes very critical public debates about atomic reactors,^1^ but also the intense and varied efforts British actors supporting the technology undertook in practice, sometimes unintentionally or even only out of necessity, to make it acceptable and to convince or cajole the public into being at least content with living in its proximity.

Some of these efforts, especially those undertaken intentionally at the national level, have already been pointed out by research (Laucht 2012; Hogg & Laucht 2012: 490–91). Focusing on one of the most important actors in that regard, the Central Electricity Generating Board (CEGB), the state-owned utility responsible for electricity supply in England and Wales from the 1950s to the 1980s, Thomas Lean and Sally Horrocks have argued that the industry engaged broadly with the public, locally as well as nationally, employing a number of “direct and indirect” methods, especially so by utilizing, based upon a “nuanced understanding of the knowledge practices of ordinary people,” “an appetite among ordinary people for knowledge based on personal experience” (Lean & Horrocks 2017: 2); and that it intensified its efforts from the mid-1970s, in the face of a number of challenges arising on the national stage (ibid.: 7–8; Rüdig 1986: 371).

In this paper, I do not focus on the CEGB’s overarching strategy or national activities as such, but employ an analysis of local public debates about nuclear power in a community neighboring nuclear reactors to further concretize, complement, and partially challenge these observations.^2^ Specifically, I turn to the history of the nuclear power plant in Oldbury-on-Severn from the late 1950s, when the plan to construct a nuclear power plant on the banks of the Severn there was first formulated, to the late 1980s, when the British government declared a moratorium on nuclear power’s further expansion (Hill 2013: 339–45; Götter 2026a: 221).

The public debates in and around Oldbury—the critique levelled at the power plant, and the measures taken to allay it—are analyzed primarily based on newspaper reports, letters to the editor, and advertisements in the *Thornbury Gazette*, the locally dominant newspaper, which, for the decade following the local government reform of 1974, took on the title of *Northavon Gazette*. Covering events relevant to the local communities, like council and committee meetings as well as activists’ protests, from high-profile events to the worries of individuals turning to the paper and its readers in search of like-minded support or to engage with differently minded people, the local newspaper allows for a rather thick description (Geertz 1991) of the local, historical discourse (Landwehr 2008) about nuclear power. Of course, one must keep in mind that it was, for most of the time, easier for the technology’s official proponents to make themselves heard than it was for its critics. While the latter often had to resort to letters to the editor, the former tended to be interviewed or featured in articles of some type or another. Nonetheless, both sides of the dispute were able to partake in the public discourse via the local newspaper’s pages. However, it is important to consider a newspaper not only as a forum for contributions to the local debate but also as a contributor to them, even though individual journalists were, in most cases, not identifiable. In sum, though, the paper took a position on various topics within the debate, adapting it to historical events and, in all probability, fluctuations in its personnel—although there were scant indications of instances of the latter influencing the fundamental stance of the *Gazette* as regards nuclear power. In addition to the local paper, additional documents from local, but also national archives, not least council protocols, documents from the licensing procedures, and press cuttings, were available to help reconstruct the historical development of the public debates on the spot. Beyond facilitating an analysis of the nuclear industry’s practices in promoting its and its technology’s acceptance, these sources also allow for a tentative analysis of the local reception of the measures taken.

The analysis shows that the CEGB was confronted with rather broad and multifaceted criticism when it presented the initial plan for a nuclear power plant near Oldbury—which, one must bear in mind, was nevertheless not as broad as the criticism levelled at some of the later nuclear power plant projects in Britain, especially when adapted American pressurized water technology was to be used or when the government seemed less willing to involve the public in the decision-making processes (Kalmbach 2021: 51; Götter 2026b: 255–56); or even elsewhere, for example in the famous German case of Wyhl (Tompkins 2016; Frickel-Pohl 2019; Milder 2019). Reacting to that, the board’s local, regional, and national representatives as well as the United Kingdom Atomic Energy Authority employed a set of measures—partly already in place because of the proximity of another, older nuclear power plant at Berkeley—aimed at overcoming its project’s headwind by making the technology and its local manifestation in the form of the Oldbury reactors acceptable to the public. Specifically, the industry strived for its reactors’ implicit acceptance—which, in this situation, but also throughout, primarily meant its perception of them as safe and necessary^3^—by measures aimed to make them seem to be, as I would call it, accountable, transparent, prestigious, and familiar. These goals were pursued by means of various individual practices, which were, in part, actively initiated, aiming for one or more of the goals, in part passively accepted, unintentionally furthering them. Other than suggested by the national perspective, however, this set of practices did not continually grow over time, with a special boost in reaction to the rising level of critique during the 1970s. It was neither a story of an ever-ongoing expansion, nor a narrative of progress, nor the flipside of a technology coming more and more under attack and being increasingly criticized. Instead, around a core group of measures consisting of radiation measurements and the early establishment of a Local Liaison Committee, the set of practices in question was flexibly adapted according to the local project’s life cycle and the state of the local and regional, much less so the national and transnational debates about nuclear power. In fact, a broad variety of practices, covering all four aimed-for aspects, was already utilized to overcome initial skepticism. The following construction phase brought with it changed priorities, as did the start of operations. With it, familiarization became very prominent, with some practices, like station visits, added to the stable core. Instead of being expanded during the 1970s, however, the efforts undertaken were toned down at the end of the decade, when local acceptance seemed secure, making the established reading of the turn to environmentally based criticism of technology seem more ambivalent (Kupper 2003), at least locally. The efforts were expanded again, and massively so, in the wake of the reactor catastrophe at Chernobyl in 1986. This event in the Soviet Union was, indeed, the only one at the transnational level clearly impacting the local strivings for nuclear power’s acceptance, which had begun in the 1950s. In sum, this indicates the importance the CEGB attached to attaining and maintaining local acceptance, which obviously necessitated and justified continuous efforts, even in calm years. Flexibly, more was done in reaction to critique or crises, with local problems, as a rule, being taken more seriously than more distant ones. In view of this flexibility on a firm foundation, then, one could say that nuclear energy was a “public technology” in terms of its acceptance, too (Trischler & Bud 2018).

The following analysis proceeds chronologically and is structured according to the developments that led to major changes in the industry’s local efforts to advocate for its own acceptance, and for that of atomic power, which happen to coincide roughly with the transitions of the decades in question.

## Preparing the Ground: The Acceptance of Berkeley Nuclear Power Station in the 1950s

Before a nuclear power station was planned at Oldbury-on-Severn, one had already been placed about seven kilometers upstream, on the banks of the Severn near Berkeley. Situated in the county of Gloucestershire, both villages belonged to the Thornbury Rural District with its local newspaper, the *Thornbury Gazette*. Consequently, measures taken regarding public acceptance of Berkeley’s power station could also be perceived in and around Oldbury, preparing the ground for the reception of the power station that came to be built there later.

The first measures taken with a view to Berkeley were aimed at overcoming two counts of criticism of the project. The first consisted of doubts about the necessity of constructing a power plant in the first place, the second of worries regarding the dangers of radioactivity. The former tended to be addressed with an appeal to a rather simple, rational argument, namely by pointing to the growing demand for electricity; the reason for the power plant being a nuclear one was then met with references to the government’s White Paper on nuclear energy from 1955, which highlighted Britain’s limited coal supplies and argued for the country’s obligation to construct nuclear reactors, if it was to remain “a leading industrial nation.”^4^ Here, in other words, transparency about the reasoning behind the plans was supplemented by a reference to what can be called national prestige (cf. Hogg 2016: 78–80). The latter criticism was met by three individual practices: for one, experts, whose judgments in technical matters were rarely persistently questioned by laypeople in the public at this point in time, publicly explained the working principles of an atomic reactor, including its multiple layers of protection, and compared its danger levels to those of established industries;^5^ and secondly, radioactivity measurements were initiated in the power plant’s vicinity so as to be able to demonstrate its (lack of) impact.^6^ While both practices aimed at transparency, the latter also presented the utility as accountable. It even contributed to its familiarization, as, for the measurements, the cooperation of local farmers was sought, in what could be seen as an early precursor of citizen (participation in) science.^7^ In any case, the procedure was explained in a way adapted to the local, rural population. One could even say that the local, agriculturally shaped practices of everyday life were revalued as controlling authorities regarding the new technology, interconnecting, in a way, technological progress and familiar practices and thereby presenting the former as trustworthy—unlike, it should be remarked, the way experts and locals famously interacted in the case of Cumbria (Wynne 1996: 62–68, 1998). In Thornbury, to be specific, Chief Construction Engineer D. Lumbard stated “that the best judge …, the most effective judge is the cow. It is the milk which is produced which is the best monitor of the radio activity in the atmosphere. … The cow is very efficient because it is sampling a large area and its product is easily obtained and quickly analysed.”^8^ Beyond aiming for laypeople’s understanding of what was going on, local experts were also briefed—and were soon to be regularly informed through a liaison committee. This had been demanded, in early 1958,^9^ by the Thornbury Rural District Council’s Medical Officer of Health, who was concerned about radiation from the power station.^10^ It was set up on September 21 the following year,^11^ actively and reliably contributing from then on to the nuclear industry’s transparency and accountability, quite concurrent with its goal of being a “good neighbour.”^12^

While Berkeley was under construction, an unintended element also accrued: the power station on the rise seemed to entail an element of the technological sublime (Nye 1996),^13^ as was often the case when nuclear power plants were constructed anywhere in the world (cf. Götter 2020: 68–69). At least, it was regularly taken up in the local news media in tones of admiration for the technical accomplishments, the sheer volume of material involved, and the skills of the personnel.^14^ While not actively brought about by the utility, the latter surely capitalized on this effect by facilitating visits by members of the public—including journalists.^15^ It was corroborated by reports about foreign experts visiting the site, marveling at the successes of British science and technology,^16^ and thereby rekindling the aura of prestige and national pride already invoked by the government’s White Paper of 1955.^17^

When, therefore, the possibility of another atomic power plant in the area came to be publicly discussed in 1958,^18^ and when the CEGB, one year later, applied for the license to construct it near Oldbury,^19^ where geological conditions were deemed well suited for the reactors and a cooling water reservoir (Götter 2024: 130), the people in and around the parish had already been targeted, intentionally as well as unintentionally, by practices aimed at establishing the transparency, accountability, familiarization, and prestige of the industry and its technology, all geared towards public perception of them as safe and necessary.

## Overcoming Initial Resistance: The Acceptance of Oldbury’s Construction in the 1960s

Consequently, some aspects of the project were accepted with minimal opposition. Concerns about radioactivity, for example, while expressed by some people,^20^ did not bother Dr. W. J. D. Cooper, the Thornbury Rural District’s Medical Officer of Health. He was satisfied that there would be no “increase” and that the region was “lucky to have the stations here.”^21^ His conviction was based upon the information that he received as a member of the liaison committee established for the Berkeley power plant,^22^ which indicates the effectiveness of the regular meetings between local officials and members of the utility^23^ in presenting the latter and their technology as accountable and transparent. The latter goal was also pursued by means of established practices, such as public talks, and by, one could say, a reified version of such explanations, an exhibition presenting “plans, maps, models and reassurances about the safety of the project” in Thornbury,^24^ when the new plans were criticized because of a lack of information about them.^25^

Another reason for resistance, however, raised by several local people, including Oldbury’s rector, Reverend D. F. Coles, concerned the fact that they had not had a say in the CEGB’s decision on Oldbury as the site for the board’s new reactor project.^26^ This aspect, the project’s accountability—and, by the way, also the demand for information—was unintentionally addressed as a consequence of the procedure for handling contested projects in the British political system: after the CEGB’s application for the construction of the power plant had been made,^27^ objections could be sent to the Minister of Power, who was to decide about it.^28^ Objections were raised and,^29^ indeed, called for by various societal actors.^30^ As a consequence of the critique surfacing in this way—of the, as one could say, crisis in acceptance—a public inquiry was initiated.^31^ This inquiry took place in Thornbury in 1960, from April 12 to 14, and on May 3. Here, proponents and opponents could state their cases in front of two inspectors. The inspectors then reported to the Minister of Power with a recommendation on how to proceed.^32^ And while the way he would decide tended to be seen as a forgone conclusion,^33^ and indeed he did rubberstamp the construction in the autumn of 1960,^34^ the fact remains that people, during the process, had been given a say. This, at least for the time being, seems to have been a suitable way to avert the festering of critique.^35^ It can even be argued that it may have motivated the CEGB to include the opinion of local political stakeholders in the future siting policy (Welsh 2000: 90; Rough 2011: 40).

This is not to imply that all objections were dispelled. Several farmers and fishermen lost their farmlands and fishing grounds and were, not surprisingly, unhappy,^36^ as were the associations representing them,^37^ especially so as their losses meant, in a few cases, the end of centuries-old ways of life.^38^ Nonetheless, the CEGB tried to smooth these blows by offering monetary compensation for their loss of income^39^ and, in the end, these losses seem to have been accepted as a price for electricity, the demand for which had been made transparent.^40^ Together with the practices already in place, then, the public inquiry seems to have contributed to increasing public acceptance of the CEGB’s reactors, when, in early 1961, the construction of the Oldbury-on-Severn nuclear power plant began.^41^

That notwithstanding, there were some misgivings during the following years. Already in 1961, for example, there was a public uproar about the radioactive emissions expected to arise during normal operation at Berkeley, with the corresponding implications for Oldbury. While some deplored the lack of transparency in this instance, which, in the eyes of the *Gazette*, was an “example of how not to ensure good public relations,”^42^ Dr. Cooper and the Local Liaison Committee felt informed about and accepted the proceedings.^43^ In light of this, the CEGB seemed satisfied that enough was being done regarding its transparency and accountability, and it made no visible attempts to actively react to the uproar. Instead, it relied on the effect of the ongoing practices to overcome the potential crisis in atomic power’s acceptance, including the regular measurements of radioactivity and reports on the results; the workings of the liaison committee, which soon took up responsibility for Oldbury in addition to Berkeley; and the prestigious framing of the power plant under construction in the local news media.^44^ The only additional action taken by the board to further the acceptance of nuclear power during this time could be seen in advertisements for electrical appliances, which suggested that these made life much easier, especially for women, while implying the necessity of electricity generation and delivery.^45^ In sum, this rather relaxed approach seems to have worked. At least, protests were discontinued, and when Berkeley was officially opened in 1963, it was generally positively regarded. Thornbury’s Rural District Council Chairman J. H. Cooke even considered it a “pleasure” to see the plant become operational and wished it a “safe and efficient operation.”^46^ The opening ceremony also provided the CEGB with an opportunity to present itself and its regional personnel to the public in several newspaper articles.^47^

This foreshadowed a new emphasis on the CEGB’s aim to present itself and its reactors as “good neighbours,” as a way to promote their acceptance. Already in the early reports about the now officially opened power plant, the personnel’s social activities were emphasized, including their founding of a “Sports and Social Club,”^48^ the “Gardening Section” of which soon started to mount a “Horticultural Show,” which attracted the regional public.^49^ Beyond these practices aimed at ingraining the power plant’s personnel in the local social fabric, the local public’s familiarization with, and consequential acceptance of the power plant itself were furthered, for the time being, by annual open days at the power plant,^50^ as well as various other visits to the site.^51^

In sum, these measures seem to have been considered rather successful locally. At least, in 1968, a commentary in the *Thornbury Gazette* stated that “people in the Severn Vale have accepted with remarkable aplomb the safety assurances which have been reiterated time and time again by the experts—assurances which have so far been justified up to the hilt.”^52^ Consequently, the official opening of the Oldbury nuclear power plant in 1969 took place against the backdrop of a widely positive view of the technology. John Spratt, chairman of Thornbury Rural District Council, surely saw it that way. He pointed out that the construction and operation of nearby Berkeley had proven the acceptability of nuclear power stations and highlighted the role of the Local Liaison Committee in keeping this up.^53^ The opening itself, which, to the journalists from the *Gazette*, seemed like “a garden-party” at the “attractive power station,” further supported this interpretation.^54^ As in the early days and during the construction phase, the plant was presented as progressive and as a boon for the nation’s greatness—in other words, as prestigious.^55^ Here, too, an open day was held to familiarize the wider public with the station.^56^

The degree to which, by the end of the 1960s, nuclear power was accepted around the new Oldbury nuclear power plant is best summed up in a comment by the local newspaper regarding an incident at a nearby chemical plant, which seemed to highlight nuclear power’s difference from other modern technologies, and confirmed the presence of one of the core elements aimed for by the industry’s practice of painting itself and its artifacts as acceptable—perception of them as safe: “We have nuclear power stations in our midst, but do not fear radioactivity because the Central Electricity Generating Board has successfully involved the public in an understanding of what is constantly being done to ensure safety.”^57^

## Succumbing to Routine: Oldbury’s Accepted Operation in the 1970s

It is against this backdrop that the practices employed to safeguard nuclear energy’s acceptability during the 1970s can be understood; during a decade, in other words, that, on the national and especially on the transnational plane, did bring several challenges to the atomic industry. These were recognizable from the start, with 1970 being “European Conservation Year,”^58^ aimed at gearing people towards nature conservation instead of expanding consumption—and the latter was evidently part of nuclear power’s appeal, as illustrated by the CEGB’s aforementioned advertisements for new electrical appliances. The issue got even more prominence when, two years later, the “Limits to Growth” report focused on the limits of the environment’s resilience (Meadows et al. 1972). A few years further down the line, the sixth report of the Standing Royal Commission on Environmental Pollution, the “Flowers Report,” highlighted nuclear power’s risks and weaknesses, especially the question of how to deal with nuclear waste and the potential danger of entering into what was called a “plutonium economy,” wherein the element would turn out to be a rather “widespread … staple commodity of energy supply” with the aimed-for construction of breeder reactors (Flowers et al. 1976, quote 73). The report, and the commission’s interest in the topic as such, had been entwined with proceedings at the Windscale nuclear facilities, which were brought to a head with an inquiry in 1977 (cf. Breach 1978; Wynne 1982).

Through it all, around Oldbury, the CEGB mostly simply continued with the practices already established to win and keep the public’s acceptance of the power plants in their neighborhood.^59^ As Oldbury Superintendent Michael Kelly put it in 1970, they wanted to “be on good terms with the public and local people.”^60^ Consequently, the boons of electrical appliances continued to be advertised,^61^ while the, not unrelated, growing demand for electricity in the wider region was highlighted.^62^ This combination provided an ongoing, implicit reasoning for the need for power stations in the first place. As regards the specific station itself, it was continuously promoted as an installation that was no danger to its surroundings: the radiation measurements taken in its vicinity were regularly presented to the Local Liaison Committee, via the local paper,^63^ and talks were given by industry experts.^64^ These practices, aiming at transparency and accountability, seem to have been quite successful. At least, in 1972, the *Gazette* observed that “the people of the Thornbury Rural District … have lost what fears they may have once had of any possible dangers from” the technology;^65^ in other words, they perceived it as harmless. Beyond that, as the paper argued, its acceptance became visible in the public’s participation in further measures undertaken by the CEGB to familiarize its power station: in everyday visits^66^ and festive open days.^67^ Moreover, the station and its personnel were often presented to the public. The plant featured in an episode of the famous “Doctor Who” television series;^68^ it won awards for tidiness;^69^ it was the stage for a boardgame championship;^70^ and from 1979 it hosted an “Annual Flower Show and Photographic Competition.”^71^ Its personnel were showcased in the local newspaper, for example, when the men at the top changed,^72^ or when staff engaged in social activities.^73^ Whether intentionally or not, aimed at this goal or not, these practices, which, in a way, downplayed almost all facets of the power plant’s “advanced” or “progressive” technology in favor of its presentation as familiar, seem to have been effective in gaining and upholding acceptance in the reactors’ neighborhood. This became clear when, in 1977, the *Gazette *countered a critique aimed at the technology with a statement that it was

“difficult to accept much of the criticism levelled against nuclear power. The men who work at Oldbury and Berkeley are not monsters bent on destroying the human race, but our neighbours and friends who have as much interest in keeping a healthy environment as we have ourselves. They have no doubts that they are employed in a very safe and healthy industry, and their confidence should be reassurance to the rest of us who cannot claim to be expert on nuclear matters.”^74^

In fact, the only instances during the 1970s in which nuclear power’s acceptance seemed endangered around Oldbury had occurred at the start of the decade, and were caused by the CEGB itself. For one, there had been minor debates about the way the power plants’ emergency plans were communicated, and nuclear waste was treated, with a lack of transparency criticized.^75^ The main challenge to nuclear energy’s acceptance, however, had come with the CEGB’s plan to build another atomic power station at Oldbury.^76^ Immediately, criticism had been raised against this plan, its environmental (health) costs,^77^ and especially a rather nontransparent proposal for chimneys for the release of radioactive air, to be constructed on top of the new station, which had been brought to the attention of the South Gloucestershire planning authority only at the last moment.^78^ This impression of inadequate transparency seems to have been at the heart of the CEGB’s trouble with the envisioned expansion, as the very transparency so far demonstrated regarding the local levels of radioactivity had motivated some in the region to accept the idea of another nuclear power plant.^79^ This impression is backed up by criticism expressed then in the *Gazette*: the CEGB had begun to answer questions in a way hardly understandable to nonexperts; and the Local Liaison Committee no longer met as frequently as it initially had—and, it was highlighted, nor did it invite the press.^80^

To counter the criticism, the CEGB explicitly pointed out that emissions had been accepted in the cases of the existing power plants and that the additional one would not be any different, with the established controls and monitoring continuing.^81^ Also, its members accepted invitations by local councilors to publicly explain the plan^82^ and, as the local newspaper put it, offered “full and frank explanations of the situation with assurance that there would be no dangers to people.”^83^

By the end of 1970, then, this resumed commitment to accountability and transparency seems to have worked, with one member of the Oldbury-on-Severn Parish Council describing the way the CEGB treated its objections as “fair and very reasonable.”^84^ Indeed, throughout most of the decade, the situation around Oldbury had seemed almost tranquil. Of course, the fact that the plans for a second station were not pursued any further and even were cancelled in 1978—when the government at Westminster decided to forgo the idea of building Steam-Generating Heavy Water Reactors and instead use Advanced Gas-cooled Reactors for the next British nuclear power stations—surely contributed to the widespread quiet.^85^ Nonetheless, it is remarkable that, while there were many critical debates on the (trans-)national level, the atomic reactors did indeed seem acceptable, locally. At least, Oldbury’s station manager, Vic Brown, was convinced that the industry had managed to win over the public, which now accepted the safety of its installations.^86^ Furthermore, the board seems to have been so convinced of its success that, at the end of the 1970s, it discontinued one of the central practices for connecting the power station and the local populace, namely the open days at the power plant.^87^

## Renewed Efforts: Oldbury’s Newly Contested Operation in the 1980s

Despite this complacency, the 1980s provided the CEGB around Oldbury with more difficult waters than the (trans-)nationally troublesome 1970s had done, resulting in renewed and stronger efforts to stimulate public acceptance of nuclear technologies, especially in reaction to two issues, one mostly local, the other stemming from the transnational level: a debate about leukemia and the catastrophe of Chernobyl.

Initially, nuclear power’s standing around Oldbury had seemed quite secure. Debates about the technology’s risks,^88^ its costs,^89^ and the plans to introduce the contested pressurized water technology to Britain,^90^ which all had some impact at the national (or even transnational) level, remained journalistic mayflies locally. Members of the regional industry publicly backed nuclear power as “one of the safest large industries in the country”^91^ and the *Gazette *was convinced that the risk of nuclear power was “an acceptable one”^92^ and that its critics “are worrying somewhat too much.”^93^ The region’s Conservative Member of Parliament, John Cope, even claimed that its people should be “proud” of the nuclear power installations in its midst.^94^ The issue of nuclear waste was taken up on a few occasions, but drew little attention overall.^95^ In fact, even protesters who criticized the sea-dumping of nuclear waste from ships sailing from the area thought their protest was not supported locally, and that people there preferred nuclear reactors to alternative means of energy generation.^96^

The industry’s efforts to promote nuclear power’s acceptability, then, seemed to be working well, despite the discontinued open days. After all, the other practices were kept up, continuously aiming to advance or at least uphold acceptance of nuclear power:^97^ its familiarization was furthered by visits to the power plant,^98^ the ongoing presence of the power station’s leadership in the local paper,^99^ and the personnel’s activities,^100^ while the flower show initiated in the late 1970s remained popular.^101^ Regarding the aspects of transparency and accountability, experts continued to give talks,^102^ information material was published,^103^ and arguments for electricity’s boons were still spread.^104^ Most importantly, the firm foundation of the CEGB’s acceptability-generating practices was still in place: radiation levels continued to be measured, and the Local Liaison Committee kept open an official channel of communication between the power plants and their neighborhood.^105^

The first serious challenge to the technology’s acceptance here was triggered by perceived clusters of childhood cancers and leukemia around nuclear installations. Initially taken up in the region in 1979, debate on the issue intensified from 1984, when the number of leukemia cases in Lydney, opposite Berkeley on the Severn’s northern shore, drew public attention and sparked a number of investigations.^106^ The CEGB’s main defense, namely the results of the continuous radioactivity measurements, which showed no “unnatural levels of radiation,”^107^ convinced at least the local paper, which called on nuclear power’s opponents “to accept the findings” and “stop the sniping.”^108^ Nevertheless, the board was willing to take a further step in the name of transparency, by supporting research into regional leukemia cases (cf. Kinlen 2011 for this debate’s further development at the national level).^109^ Once the debate had been invigorated by the reactor catastrophe at Chernobyl in the Soviet Union,^110^ the CEGB was even willing to support “independent monitoring of radiation,”^111^ an idea that had been brought up before, but was boosted by the disaster.^112^ Even in the wake of Chernobyl, it has to be remarked, numerous people defended the technology in letters to the editor of the local paper. And although it is unclear, whether at least some of the numerous letter writers worked for the CEGB, this is another indicator of the prevailing high level of local acceptance.^113^

That notwithstanding, with Peter Walker, the Secretary of State for Energy, demanding “more openness,”^114^ Chernobyl markedly boosted the CEGB’s efforts to win and safeguard nuclear power’s acceptance nationally (Kalmbach 2021: 33), as well as around Oldbury. Clearly, the lull that had crept in at the end of the 1970s ended, because, as on the national level (Brown 1992: 192–93, 199), the accident at the Soviet reactor caused a noticeable disquiet here.^115^ As a Labour politician from the nearby Bristol area put it, “The message from Chernobyl is clear. Accidents in nuclear power stations can happen. They do happen. And they have devastating effects on the lives of people for hundreds of miles around.”^116^ In one instance, the catastrophe was framed as putting “the nuclear power industry … on trial,”^117^ and an independent councilor from Chepstow, just across the Severn from Oldbury, demanded an end to the technology’s application,^118^ while skeptical Labour and SDP–Liberal Alliance politicians at least called for more transparency, especially in the form of public meetings of the Local Liaison Committee.^119^ Of course, the *Gazette* once more backed this call, publishing it time and again.^120^

The CEGB instantly reacted by pointing out that it had not been responsible for averting the demanded openness in the past. Now, it at least called for “a special meeting”^121^ and, via Keith Baddley, Oldbury’s station manager, actively invited politicians critical of atomic energy to visit the station.^122^ Further prodded by the local newspaper,^123^ the Local Liaison Committee’s meeting was indeed opened up in the following year.^124^ Going even further, in a move lauded by the *Gazette*,^125^ the CEGB then initiated a reform of the committees into Local Community Liaison Councils, which were to meet more often and to include councilors from a wider area, as well as additional members beyond councilors.^126^ Further steps to highlight its transparency and accountability included an amplification of the way the emergency plans were publicly presented. After making them available in rough outlines in 1978, now, more details were provided.^127^ Also, problems and various goings-on at the local power stations were publicized more actively,^128^ a biweekly newsletter was published from 1987,^129^ and, of course, the radiation levels in the environment continued to be measured.^130^ Besides transparency, the goal of familiarization was pursued with renewed impetus. Talks were given,^131^ and the station, its staff, and their activities continued to be showcased.^132^ Also, visits to the power station, possible throughout, were now actively advertised.^133^ The CEGB even reactivated the concept of open days, the first of which, announced by a full-page advertisement in the *Gazette*,^134^ brought about 15,000 visitors to the station,^135^ marking the reemergence of the tradition after a seven-year lapse.^136^ Beyond that, television advertisements were produced,^137^ and an advertisement campaign in local papers, launched in late 1986, aimed almost every week to explain the technology to the public, by posing and answering various questions, such as one about different sources of radiation in everyday life.^138^ One could even say that renewed attempts were made to stage the power station as prestigious, as when high-profile visits to it were publicly presented, including one by members of the International Atomic Energy Agency, reviewing Oldbury’s safety.^139^

To individual critics, the CEGB’s newfound drive to talk about itself amounted to “the biggest propaganda campaign since Germany in the second world war.”^140^ To local Conservative Member of Parliament, Cope, however, the CEGB seemed rather successful—at least, as he saw it, a majority of people in the region did accept nuclear power and, especially so, the local nuclear power plants, the staff of which, in his view, were “extremely frank about their problems” and did not hide them.^141^ Fittingly, when Berkeley was closed down at the end of the 1980s, many locals were sad to see it go, or so noted the *Thornbury Gazette*:^142^ “What we can certainly say is that Berkeley has been a good neighbour to us and has had a significant role to play in all our lives. Its retirement is sad but Berkeley will not be forgotten, that is for sure.”^143^ In this sense, then, the CEGB’s efforts to promote acceptance of nuclear power around Oldbury had been successful to an overwhelming degree.

## Conclusion: Technology Acceptance following Flexible Measures on a Firm Foundation

The CEGB, in short, used a variety of individual practices to demonstrate its own and nuclear power’s acceptability, flexibly adopting them to the local situation and perceived necessities. Appealing at the same time to “hearts and minds,” it demonstrated accountability and transparency from the start, tried to familiarize the local population with the technology, and highlighted the latter’s prestige. The unintended, one-off public inquiry, which was part of the British political system, surely contributed to the former two, as did the institution of the Local Liaison Committee and the regular radiation measurements. These two came to represent the firm foundation for proving the accountability and transparency of the board and its reactors for the whole period under consideration here. They represented, in other words, the basic set of practices aimed at making the atomic artifact perceptible as a safe and acceptable neighbor. During the construction phases, then, the prestige of British nuclear technology was especially prominent, while the public’s familiarization both with it and the people operating it came to the fore once the power plants had become operational. It is especially remarkable that the ongoing measures on site were simply kept up throughout most of the 1970s, a decade when the (trans-)national debate turned critical. The established and ongoing local practices to present the power plant as acceptable were, obviously, deemed sufficient to weather the more distant storms facing the technology. An intensification of the local efforts was only visible at the start of the decade, after the CEGB had proposed another power plant in Oldbury. At its end, and in view of the cancellation of these expansion plans, the open days, a central measure aimed at familiarizing the population with the technology, were even phased out. In the Oldbury area, a substantial change in the CEGB’s attempts to win the people’s acceptance of it and its installations was brought about only in 1986, when the Chernobyl catastrophe spurred criticism of the technology locally as well as (trans-)nationally. Now, amongst various other measures aimed at familiarization, the open days were reinstated, in a bid to (again) convince the local population that the CEGB and its power plants were “good neighbours.” In the same vein, the fundamental, ongoing practices aimed at transparency and accountability were strengthened and supplemented by new ones. In the end, in a way, even attempts at staging the local power plant as prestigious were resumed.

This analysis, then, was able to demonstrate that, to make nuclear power acceptable in the vicinity of a power plant, the nuclear industry, most prominently the CEGB, constantly strove to highlight its and its technology’s transparency, accountability, and prestige, and to familiarize the local population with them. The individual practices aimed at these goals were primarily adjusted to the local debates and the phases in the local artifact’s life cycle, rather than to (trans-)national events. The one striking exception was the Chernobyl catastrophe, which triggered an unprecedented broadening and intensification of the measures taken, and even a return to certain practices that had been deemed no longer necessary for years. The local perspective taken up here, then, demonstrates that the CEGB established a firm foundation of measures that it relied upon throughout to assure its and its power plants’ acceptability. Beyond that, in phases of higher demand, but also as a sometimes, unintended consequence of other activities, further steps were flexibly taken. The perspective taken here, in other words, is conducive to a concretization and partial correction of the current state of research regarding the practices employed to produce acceptance of nuclear power, focused on the national level.^144^ Also, it allows for sideways glances at the consequences of the practices employed; or at least at their attributed successes or failures as instruments of social engineering. In this regard, it can be stated that, overall, they were regarded as successes, and the nuclear reactors were accepted as “good neighbours.”^145^

## Footnotes

1 These are at the center of a research project undertaken by Christian Götter and funded by the German Federal Ministry of Education and Research (Funding Reference Number 01UL1706X), as part of the funding format “Kleine Fächer – Große Potentiale.”

2 The need to study the local when aiming to understand “nuclear culture” was highlighted by Hughes (2012).

3 The interplay of the perceived risks and the perceived (economic) necessity of nuclear power was identified as crucial for its acceptance early on by social scientists: Renn (1981); for the importance of “health and safety”: Mills & Williams (1986: 420).

4 A Programme of Nuclear Power, February 1955 (draft), Paragraph 21, p. 5, National Archives, London (NA) CAB 129/73/c/55/31/31; cf. ibid., Paragraphs 2 and 3, p. 1, for the relative importance attached to the arguments regarding the country’s scientific and technical prowess and its supply situation regarding conventional sources of energy.

5 For a presentation by engineer Philip Whitelock in Berkeley’s Primary School, cf. Nuclear Energy. What is Planned at Berkeley? One Lorry Load of ‘Fuel’ Each Week. *Thornbury Gazette* (*TG*) (04.05.1957); for one at nearby Dursley’s Rotary Club: Power Station. Why Berkeley Site was Selected. C.E.A. Engineer Gives Details of Vast Undertaking. *TG* (29.06.1957); for one at the Old Thornburian Society: Old Thornburian’s Dinner. Nuclear Power and its Link with Milk. *TG* (19.07.1958).

6 Radio-Activity on Plant and Animal Life. Research Assurance Sought by Farmers. *TG *(26.04.1958); these measurements were initiated by the United Kingdom Atomic Energy Authority (UKAEA).

7 Test Sites for Atomic Radiation. *TG* (17.05.1958).

8 Old Thornburian’s Dinner. Nuclear Power and its Link with Milk. *TG* (19.07.1958).

9 Atomic Station Liaison. Exploratory Meeting to be Held. *TG *(13.12.1958).

10 Clean Air and Radiation. Two Subjects of Importance. *TG *(22.11.1958).

11 Berkeley Liaison Committee. The Citizen (*TC*) (07.01.1959); The Local Liaison Committee. *TG *(06.04.1963).

12 Good Relations Between Site and Town. Berkeley Residents Outnumbered by Employees. *TG* (07.02.1959).

13 Cf., for the positive connotation of technological progress, even beyond the time and place in focus: Segal (1985).

14 Cf. Atomic Station, in: *TG* (01.06.1957).

15 Scouts Visit Power Station. *TG* (27.07.1957); Nerve Centre of Power Station. Visitor Finds Himself Bewildered. *TG *(09.01.1960).

16 Japan’s Interest in Nuclear Power. Commissioner Impressed by Berkeley Progress. *TG *(16.11.1957).

17 Cf. Berkeley is a Tonic to our Industry. *TC *(21.06.1958); Power Station Work is up to Schedule. Two Reactors and Turbine Hall Almost Complete. Plant Installation now in Progress. *TG* (07.02.1959). Later, in the debate about Oldbury, some critics allegedly forewent their stance because the power plant’s necessity was argued for with a view to national considerations: Do Nuclear Power Stations Mean Danger? Pertinent Question to Inspector at Thornbury Inquiry. *TG *(23.04.1960).

18 In The Estuary. Second Nuclear Station. *TG* (17.05.1958).

19 Second Nuclear Station on Severn Bank. Application To Develop 175-Acre Site. *TG* (28.03.1959).

20 Critical voices in this regard were raised in Oldbury Parish Council, which also highlighted the loss of farmland and fishing grounds: Parish’s Nuclear Protest. *TG* (02.05.1959); critics were also present in Thornbury Rural District Council: Nuclear Station. Council Told 90 Per Cent of Oldbury People Are Opposed. *TG* (02.05.1959).

21 Oldbury’s Reaction to New Power Station. Farmers and Fishermen Perturbed but – ‘It Has Got to Come.’ *TG *(04.04.1959).

22 Except in the Event of War. No Danger Envisaged from Radiation. *TG* (11.04.1959); cf. To Protect the Public. Steps Being Taken at Berkeley. *TG* (26.03.1960).

23 Berkeley Power Station. Liaison Committee Meets. *TG* (22.10.1960).

24 New Power Station. Biggest in the World. Work Planned to Start in 1965. 350 Acre Reservoir in River Severn. *TG* (09.05.1959); Impact On The Local Community. *TG* (14.06.1969); cf., for public discussions in councils: Nuclear Station. Council Told 90 Per Cent of Oldbury People Are Opposed. *TG* (02.05.1959).

25 Perturbed: More Information Needed. *TG* (02.05.1959).

26 Oldbury’s Reaction to New Power Station. Farmers and Fishermen Perturbed but – ‘It Has Got to Come.’ *TG* (04.04.1959); Amor Patriae: Oldbury Power Station. *TG *(11.04.1959).

27 Central Electricity Generating Board: Public Notice. *TG* (02.05.1959).

28 Ibid.

29 Cf. Nuclear Station Objection. Not Prudent in View of Unknown Effects. *TG* (23.05.1959); Vale Power Stations. Thornbury Objection Received. *TG* (13.06.1959); Second Station. Hundreds Sign Petition. *TG *(25.07.1959).

30 Cf. Ryder, T. A., Chairman, County Branch, C.P.R.E., Cam., Glos. A Lone Voice. *TG *(16.05.1959); Davidson, Brian (Berkeley). Nuclear Power Station at Oldbury. *TG* (16.05.1959).

31 Second Power Station. Objections Are Modified. *TG *(02.01.1960).

32 Report to the Minister of Power, 04.07.1960, NA POWE 14/1249; Oldbury nuclear station. Inquiry in April. *TC *(16.03.1960); 385 Will Be Employed at Second Power Station. Inquiry Hears Objections to Board’s Proposal. *TG* (16.04.1960).

33 Cf. A Lone Voice. *TG* (09.05.1959); this accusation against public inquiries regarding the nuclear industry was rather widespread among its critics, cf. Cutler & Edwards (1988: 180).

34 Minister approves new Glo’shire atom station. Work likely to begin soon at Oldbury. *TC* (20.10.1960).

35 Besides, this can be seen as another indicator of nuclear energy being a “public technology,” cf. Trischler & Bud (2018).

36 Oldbury’s Reaction to New Power Station. Farmers and Fishermen Perturbed but – ‘It Has Got to Come.’ *TG* (04.04.1959).

37 Power Station Opposed. Effect On Salmon Fishing. *TG* (16.05.1959).

38 Oldbury’s Reaction to New Power Station. Farmers and Fishermen Perturbed but – ‘It Has Got to Come.’ *TG* (04.04.1959).

39 Report to the Minister of Power, 04.07.1960, pp. 51, 67, NA POWE 14/1249; cf. 385 Will Be Employed at Second Power Station. Inquiry Hears Objections to Board’s Proposal. *TG* (16.04.1960); Götter (2024: 130–32).

40 New Power Station. Biggest in the World. Work Planned to Start in 1965. 350 Acre Reservoir in River Severn. *TG *(09.05.1959).

41 Most Powerful Nuclear Potential in the World. Work on the New Severn Atom Station this Month. *TC* (10.01.1961).

42 Nuclear Station Wastes. Was News of Discharge Concealed? *TG* (21.01.1961); quote: 1955 and after. *TG *(28.01.1961).

43 Radioactive Waste. Strong Protest at Dursley. *TG* (14.01.1961); Nuclear Station Wastes. Was News of Discharge Concealed? *TG* (21.01.1961); for Oldbury, cf. Into Water or Air? Nuclear Discharge Wastes. *TG *(13.05.1961).

44 Cf., for the first aspect: Berkeley Power Station Construction Cost £ 46-Million. *TG *(23.06.1962); Radioactivity Increase Not Due to Nuclear Power Station. *TG* (16.03.1963); Nuclear Power at Oldbury and Berkeley. *TG* (29.10.1966); Radiation Levels. Monitoring in the Vale. *TG* (06.04.1968); for the second: Prepared for an Emergency. Assurances From Power Station Officials. *TG* (27.10.1962); Topics Discussed by Double Gloster. Power Stations.* TG *(28.03.1964); Public and the Power Stations. *TG* (19.10.1968); for the last: Nuclear Power Station at Oldbury Fast Becoming a Reality. *TG* (23.09.1961); Power Station Progress at Oldbury. Project Is Approaching Half Way Stage. *TG* (21.03.1964); Progress at Oldbury. Civil Engineering Almost Complete. *TG* (30.01.1965); Oldbury Power Station Construction Prompts Awe and Wonder. *TG* (06.02.1965); Phillips, H. W.: Severnside’s Attractive Giant. Oldbury Progress. *TG* (09.03.1968).

45 Advertisement: She can’t have one without the other. *TG* (08.07.1961); for an analysis of advertisements for electrical washing machines and the image of women evoked hereby in Germany, see Weber (1998).

46 Cooke, J. C. [*sic*]: Good Wishes from Thornbury RDC. *TG *(06.04.1963).

47 The Men Behind Berkeley. *TG* (06.04.1963); The Men of Berkeley. Ibid; Success Stories from CEGB Training. Ibid.

48 They’re Settling Down Well. *TG* (06.04.1963).

49 Berkeley Nuclear Sports and Social Club. Gardening Section Organises Horticultural Show. *TG* (21.09.1963).

50 More Power from Power Station at Berkeley for the Winter. *TG* (24.10.1964); picture in *TG* (25.06.1966); Thousands of People Have Visited Berkeley Power Station. *TG* (29.07.1967).

51 Cf. picture in *TG* (10.07.1965); No Danger from Nuclear Power Station. *TG* (11.03.1967); allowing (certain) parts of the public to visit a site was a rather well-established concept with the electricity industry—Siemens and AEG had already been active in this regard in the 19th century: Zipfel (1997: 50, 153–54); for the touristic context of this practice in general: Mysliwietz-Fleiss (2022: 239–54).

52 County Comment by Double Glo’ster: Nuclear Safety. *TG* (10.02.1968).

53 Statement by John Spratt. *TG* (14.06.1969).

54 ‘Garden Party’ Send Off for Oldbury Power Station. *TG* (14.06.1969).

55 Oldbury Opening. New Features In £ 60 M Project. *TG* (14.06.1969); Oldbury Opening, commentary by Station Superintendent Michael Kelly. *TG* (14.06.1969).

56 Open Day for The General Public. *TG* (14.06.1969).

57 Arsenic and Old Lace. *TG* (06.12.1969).

58 County Comment by Double Glo’ster: Pollution. *TG* (07.02.1970).

59 It should be remarked, however, that the “plutonium economy” was debated locally, cf. letters to the editor in the *Gazette* during the weeks from 15.10.1977, e.g. Peter Brown (Dursley): Nuclear Power. *Northavon Gazette *(*NG*) (12.11.1977); John Marjoram (Stroud): Nuclear Power. *NG* (26.12.1977).

60 Local Power Stations Did a Good Job. *TG* (19.12.1970).

61 Cf. *TG* (30.05.1970); Electricity for Home Heating. *TG* (26.08.1972); Dursley Powers Electric Cars. *NG* (05.07.1975); advertisement in *NG* (07.10.1978).

62 Cf. Midlands Electricity Board Reports £ 4.9 M. Surplus. *TG* (17.10.1970); Good Record. *TG* (08.09.1973).

63 Oldbury Power Station. *TG* (25.03.1972); Nuclear Power Stations Good Record. *TG* (23.03.1974); Nuclear Power in the Vale. *NG* (29.03.1975); Open Days at Power Stations. *NG* (30.04.1977); Emergency Plan for the Nuclear Power Stations. *NG* (29.04.1978).

64 Nuclear Safety. *NG* (29.09.1979).

65 Quote: 3,000 attracted to Oldbury’s open day. *TG* (26.08.1972).

66 Oldbury Concern on Power Plan and Flooding. *TG* (06.06.1970); cf. Pratchett, Terry (Press Officer, CEGB): Always Open. *TG* (31.10.1986).

67 Open Day and Fete at Oldbury Nuclear Power Station. *TG* (11.09.1971); Open Day at Power Station. *TG* (12.08.1972); County Comment by Double Glo’ster: Berkeley Power Station. *TG* (12.05.1973); Open Days at Power Stations. *NG* (30.04.1977).

68 Dr Who and Thornbury Adventure. *NG* (02.10.1976).

69 Power Plus Clean Neat Surrounds. *NG* (12.11.1977).

70 It also Produces Power. *NG* (19.02.1977).

71 Produce Show at Oldbury on Severn. *NG* (15.09.1979).

72 New Supers at Power Stations. *TG* (24.03.1973); Brown, V. (Station Manager, Oldbury): Reliable Power. *NG* (10.09.1977); A Miner’s Son with Nuclear Faith. *NG* (29.10.1977).

73 Oldbury Fire Fighters in Good Form. *TG* (23.09.1972).

74 Nuclear Power. *NG* (22.10.1977).

75 Cf. Assurances Wanted on Power Station Safety. *TG* (01.05.1971); Risk Exists, but it is Remote. CEGB Assurance to Thornbury RDC on Power Stations. *TG* (05.06.1971); Age of Protest. *TG* (08.05.1971); Radio Active Waste Disposal from the Power Stations. *TG* (08.03.1969); Pollution. *TG* (02.10.1971).

76 Nuclear Power. Stations In Estuary. *TG* (14.02.1970).

77 Cf. Minutes, Meeting, Public Works and Planning Committee of the Thornbury Rural District Council, 06.03.1970, p. 461, in: Gloucestershire Archives, DA38/100/34; Scare Stories of Nuclear Tombs at Severnside. *TG* (14.03.1970); A Radioactive Air Discharge from Oldbury ‘B’? *TG* (25.04.1970); Worry Over Nuclear Build Up. Thornbury RDC to Press for Meeting with the Experts. *TG* (30.05.1970); Nuclear Concern. *TG* (30.05.1970); Oldbury Concern on Power Plan and Flooding. *TG* (06.06.1970); Criminal Folly of Pollution Menace. *TG* (04.07.1970); Opposed to Second Station. *TG* (19.09.1970).

78 A Radioactive Air Discharge from Oldbury ‘B’? *TG* (25.04.1970).

79 Scare Stories of Nuclear Tombs at Severnside. *TG* (14.03.1970).

80 Nuclear Concern. *TG* (30.05.1970); Severnside Safety. *TG* (05.06.1971).

81 A Radioactive Air Discharge from Oldbury ‘B’? *TG* (25.04.1970); Question and Answer About Nuclear Power Station Safety. *TG* (20.06.1970).

82 Worry Over Nuclear Build Up. Thornbury RDC to Press for Meeting with the Experts. *TG* (30.05.1970); RDC and Nuclear Experts. *TG* (06.06.1970).

83 Question and Answer About Nuclear Power Station Safety. *TG* (20.06.1970).

84 Oldbury Protest Draws Assurance from Generating Board. *TG* (28.11.1970).

85 Oldbury Power Station. *NG* (29.07.1978).

86 A Miner’s Son with Nuclear Faith. *NG* (29.10.1977).

87 Pratchett, Terry (Press Officer, CEGB): Always Open. *TG* (31.10.1986).

88 Politics and the Nuclear Debate. *NG* (28.06.1980).

89 Ibid.

90 Nuclear Power. *NG* (22.03.1980).

91 Campaign Against Nuclear Power in S. West. *NG* (29.03.1980).

92 Taking Risks. *NG* (21.03.1981).

93 Better Safe … *NG* (18.06.1983).

94 Reasons for Being Proud of Local Nuclear Power. *NG* (28.11.1981).

95 Cf. Dellbridge, Olivia (Berkeley): Nuclear Wastes. *NG* (18.07.1981); Brownlow, Howard (Sharpness): Nuclear Waste. *NG* (25.07.1981); Green, L. (Miss) (Berkeley): Nuclear Waste. *NG* (25.07.1981); Human Error Blamed for Radioactive ‘Leak.’ *NG* (21.11.1981); Oldbury Error Led to Leak of Radiation. *NG* (21.11.1981); Big Police Presence, and Low Key Protests as ‘Gem’ Loads. *NG* (07.08.1982).

96 Big Police Presence, and Low Key Protests as ‘Gem’ Loads. *NG* (07.08.1982).

97 I am indebted to Fabian Zimmer for this wording.

98 Pratchett, Terry (Press Officer, CEGB): Always Open. *TG* (31.10.1986).

99 Changes at two Nuclear Power Stations. *NG* (28.08.1982); New Manager at Oldbury. *NG* (26.08.1983); New Chief for Local Power Station. *TG* (04.04.1986).

100 Power Men’s £ 1,000 Gift for Handicapped Youngsters. *NG* (21.05.1983).

101 Station Show was a Keen Affair. *NG* (30.09.1983); Flower Power on Show. *TG* (20.09.1985).

102 Debate on Nuclear Power. *NG* (09.02.1980); cf. Edmondson, B. (Director, Berkeley Nuclear Laboratories): Nuclear Power. *NG* (22.03.1980); Nuclear Power Safety. *NG* (05.04.1980); Nuclear Sniping. *TG* (21.09.1984).

103 Fogg, Brian (Public Relations Officer, CEGB, Generation Development and Construction Division): Nuclear Power. *NG* (12.04.1980).

104 In this case, by the UKAEA: Nuclear in Life and Leisure. *NG* (06.07.1984).

105 Nuclear Power. Discharge Control. *NG* (03.05.1980); Safety Reports on Nuclear Stations. *NG* (01.05.1982); Nuclear but Negligible Radiological Impact. *NG* (23.04.1983); Safety Check Finds Nothing Over 25 Years of Activity. *NG* (04.06.1983); N‑stations Have a ‘negligible effect.’ *NG* (27.04.1984); N‑plants all Clear in Safety Checks. *NG* (29.06.1984); No Probe into N‑plants. *TG* (26.04.1985); Nuclear Power Watchdogs Stay ‘secret.’ *TG* (25.04.1986).

106 Cf. Pearson, Trish and Martin (Dursley): Nuclear Wastes. *NG* (14.07.1979); Brown, Mary (Cam Green): Nuclear Power. *NG* (01.03.1980); Brown, Peter (Dursley): Nuclear Power. *NG* (15.03.1980); Williams, Alan Bryn (Thornbury): Nuclear Controversy Futile. *NG* (16.03.1984); Cash Appeal for N‑Plant Investigation. *NG* (22.06.1984); Leukaemia Link Probe. *TG* (09.11.1984); No Probe into N‑Plants. *TG* (26.04.1985); Nuclear Industry ‘too secretive.’ *TG* (25.04.1986).

107 N‑Plants all Clear in Safety Checks. *NG* (29.06.1984).

108 Nuclear Sniping. *TG* (21.09.1984).

109 Hall, Richard: N‑Plant Cancer Fears ‘groundless.’ *TG* (22.03.1985).

110 Post-Chernobyl … … Debate Rages on. *TG* (06.06.1986); Council Storm over Leukaemia ‘dismissal.’ *TG* (04.07.1986); Philp, Gerry (District Councillor, Alliance, Chipping Sodbury): Link Between Deaths yet to Be Proved. *TG* (11.07.1986); Boreham, Dr. D. P., Dr. H. K. Chadha (Elberton): Nuclear Power will Cost Us the Earth. *TG* (07.11.1986).

111 Quote: Call for Independent Radiation Monitoring. *TG* (31.10.1986); Radiation Checks Plan in the West. *TG* (07.11.1986); cf. Mizen, Alec (Buckover): Extra Monitoring will Be Costly. *TG* (09.01.1987); Radiation Monitoring Move. *TG* (20.02.1987); First Liaison Meeting Open to Press. Monitoring Service is Welcome. *TG* (24.04.1987); Snub for New Radiation Lab. *TG* (22.05.1987).

112 No Probe into N‑Plants. *TG* (26.04.1985).

113 Cf. Davies, B. S. J. (Uley): Muddled. *TG* (14.11.1986); Mizen, A. W. (Buckover Bird Sanctuary): Muddled. *TG* (14.11.1986); Willcox, B. W. (Olveston): Muddled. *TG* (14.11.1986); Clapham, Valerie (Oldbury-on-Severn): Man’s Environment is Naturally Hostile. *TG* (21.11.1986); Gill, W. (Thornbury): No Proof of a Radiation Link. *TG* (21.11.1986).

114 Allison, Hilary: Emergency Plans Open to Public. Power Stations’ Bid to Dispel Secrecy. *TG* (17.10.1986); this demand, then, was not new when it was published, two years later, as one result of the considerations of The Watt Committee on Energy, cf. Lewis (1988).

115 Chernobyl: Avon Call for Report on Disaster. *TG* (09.05.1986); cf. Farmers’ Need of Nuclear Guidelines. *TG* (25.03.1988).

116 Danger just too Great? *TG* (30.05.1986).

117 Chernobyl ‘Demands Honesty.’ *TG* (16.05.1986).

118 Fleming, Pat (Gwent): Time to Call a Halt Now? *TG* (30.05.1986).

119 Candidates Condemn N‑Plant Secrecy. Call to Reassure Public. *TG* (30.05.1986); cf. Chernobyl ‘Demands Honesty.’ *TG* (16.05.1986); Candidates Condemn N‑Plant Secrecy. Call to Reassure Public. *TG* (30.05.1986).

120 Our Point of View. *TG* (23.05.1986).

121 Candidates Condemn N‑Plant Secrecy. Call to Reassure Public. *TG* (30.05.1986).

122 Labour Candidates Tour Oldbury Nuclear Power Station. Emergency Plan Talk Invitation. *TG* (04.07.1986).

123 Our Point of View. *TG* (17.10.1986).

124 First Liaison Meeting Open to Press. Monitoring Service is Welcome. *TG* (24.04.1987).

125 Our Point of View. *TG* (15.01.1988).

126 CEGB Plan Shake-up in N‑Plant Links. *TG* (03.07.1987); Propaganda Claim Against CEGB. *TG* (23.10.1987); Our Point of View. *TG* (15.01.1988).

127 Our Point of View. *TG* (17.10.1986); Allison, Hilary: Emergency Plans Open to Public. Power Stations’ Bid to Dispel Secrecy. *TG* (17.10.1986).

128 ‘Lessons to be learnt’ from Nuclear Blast. *TG* (31.10.1986); N‑Train Derailed ‘but no danger.’ *TG* (06.02.1987); Reactor Briefly Shut. *TG* (07.08.1987); Detector Set off. *TG* (14.08.1987); Oldbury Turbine Shutdown Averted. *TG* (09.10.1987); Noisy Finale at Oldbury. *TG* (09.06.1989).

129 Our Point of View. *TG* (05.06.1987).

130 Mizen, A. W. (Buckover Bird Sanctuary): Muddled. *TG* (14.11.1986); Mizen, Alec (Buckover): Extra Monitoring Will Be Costly. *TG* (09.01.1987).

131 Watson, Neil: No Chernobyl in Northavon, Meeting Told. *TG* (12.09.1986).

132 Power Station Man is Honoured by SJAB. *TG* (22.05.1987); World Energy Film Shot at Oldbury. *TG* (18.09.1987); Oldbury Holds Muster ‘Demo.’ *TG* (18.09.1987); Changes at Oldbury Power Plant. *TG* (15.04.1988); CEGB’s Five in Paris. *TG* (03.06.1988); Power Station Celebrates 21st Birthday. *TG* (30.09.1988); Oldbury Finalist. *TG* (04.08.1989).

133 Cf. An Open Invitation to Anyone who Wants to Know About Nuclear Power and Radiation. *TG* (21.11.1986); An Open Invitation to Anyone who Wants to Know About Nuclear Power and Radiation. *TG* (09.01.1987); What Goes on Inside Oldbury? *TG* (06.03.1987); Business as Usual. *TG* (22.07.1988).

134 Visit a POWER STATION. *TG* (05.09.1986); Visit a POWER STATION. *TG* (12.09.1986).

135 Pearce, Rosie: Thousands at Power Station Open Days. *TG* (19.09.1986).

136 Campsite Within Power Station. *TG* (23.09.1988); Pointing, R. (Plant Operator, Thornbury): Unfair Way to Treat the Disabled. *TG* (21.10.1988).

137 Compton, Pam and Rob (Charfield): What Does it Prove? *TG* (03.10.1986).

138 Taking Mystery out of N‑Power. *TG* (21.11.1986); Radiation is Everywhere. Where do You Think it Comes from. *TG* (12.05.1986); cf. If You Question Our Safety Expertise, Come and Question Our Safety Experts. *TG* (28.11.1986); What We Monitor Tells Us that Oldbury Power Station Hasn’t Endangered the Local Environment. *TG* (12.12.1986); It’s no Accident that the British Nuclear Power Industry is One of the Safest in the World. *TG* (23.01.1987); Why the British Nuclear Power Industry is Amongst the Safest in the World. *TG* (24.04.1987); An Open Letter from the CEGB to Put Nuclear Power into Perspective. *TG* (16.09.1988); Radiation. It’s a fact of life. *TG* (23.09.1988); Our Operators are Highly Qualified and Fully Trained, and We Have Fail-Safe Safety Systems at Our Nuclear Power Stations. *TG* (25.11.1988); An Open Letter from the CEGB to Put Nuclear Power into Perspective. *TG* (13.01.1989).

139 Russians on Oldbury Visit. *TG* (11.12.1987); Visit to Oldbury. *TG* (24.06.1988); Campsite Within Power Station. *TG* (23.09.1988); World Team Praises Safety at Oldbury. *TG* (28.07.1989).

140 Propaganda Claim Against CEGB. *TG* (23.10.1987).

141 Fahey, Kevin: N‑Plant Neighbours May Be Compensated. *TG* (24.04.1987).

142 Our Point of View. *TG* (15.07.1988); Our Point of View. *TG* (03.03.1989); The Final Switch Off. *TG* (31.03.1989); Green, Ros: Berkeley—The Final Shutdown. Power Worth £ 1.5 Billion. *TG* (07.04.1989).

143 Beginning of End for Berkeley. *TG* (31.03.1989).

144 Proving Joachim Radkau’s dictum that historians of technology should pay more attention to the specific situations in which technologies were applied: Radkau (2008: 9).

145 For their critical and constructive feedback, I am indebted to Fabian Lampe, Sebastian Mönnich, and Heike Weber.
